# PdNPs/NiNWs as a welding tool for the synthesis of polyfluorene derivatives by Suzuki polycondensation under microwave radiation

**DOI:** 10.1038/s41598-024-52795-w

**Published:** 2024-01-28

**Authors:** Tomasz Wasiak, Dominik Just, Andrzej Dzienia, Dariusz Łukowiec, Stanisław Wacławek, Anna Mielańczyk, Sonika Kodan, Ananya Bansal, Ramesh Chandra, Dawid Janas

**Affiliations:** 1 Department of Chemistry, Silesian University of Technology, B. Krzywoustego 4, 44-100 Gliwice, Poland; 2 Materials Research Laboratory, Faculty of Mechanical Engineering, Silesian University of Technology, Konarskiego 18a, 44-100 Gliwice, Poland; 3 Institute for Nanomaterials, Advanced Technologies and Innovation, Technical University of Liberec, Studentská 1402/2, 461 17 Liberec 1, Czech Republic; 4 Nanoscience Laboratory, Institute Instrumentation Centre, Indian Institute of Technology Roorkee, Roorkee, 247667 India

**Keywords:** Carbon nanotubes and fullerenes, Conjugated polymers

## Abstract

Conjugated polymers are promising tools to differentiate various types of semiconducting single-walled carbon nanotubes (s-SWCNTs). However, their synthesis is challenging. Insufficient control over molecular weights, and unpredictive/unrepeatable batches hinder possible applications and scale-up. Furthermore, commercial homogeneous catalysts often require inert conditions and are almost impossible to recycle. To overcome these problems, we present a nanocatalyst consisting of magnetic nickel nanowires decorated with highly active palladium nanoparticles. A two-step wet chemical reduction protocol with the assistance of sonochemistry was employed to obtain a heterogeneous catalyst capable of conducting step-growth Suzuki polycondensation of a fluorene-based monomer. Additionally, we enhanced the performance of our catalytic system via controlled microwave irradiation, which significantly shortened the reaction time from 3 d to only 1 h. We studied the influence of the main process parameters on the yield and polymer chain length to gain insight into phenomena occurring in the presence of metallic species under microwave irradiation. Finally, the produced polymers were used to extract specific s-SWCNTs by conjugated polymer extraction to validate their utility.

## Introduction

Polymers are one of the most diverse and versatile groups of chemical compounds. Recently, π-conjugated polymers have emerged as highly electroactive and photoactive materials, which can be used to craft various electronic devices^1,2^. Most of their applications involve photovoltaics^3,4^, memory devices, or organic light-emitting diodes (OLEDs)^5,6^. However, they are considered almost indispensable in the field of chirality selective sorting of single-walled carbon nanotubes (SWCNTs).

SWCNTs are produced by various methods such as arc discharge (AD)^7^, pulsed laser vaporization (PLV)^8^, and chemical vapor deposition (CVD)^9^, to name a few. Unfortunately, among these approaches, the final product is obtained as a mixture of SWCNTs of different types (both metallic and semiconducting), which greatly reduces their research diversity and implementation potential. Therefore, these raw materials must be subsequently processed to isolate SWCNTs of individual types. Currently, a spectrum of methods is available for SWCNT purification^10^. One of the most convenient and effective techniques is conjugated polymer extraction (CPE). Polymers readily deposit on the SWCNT surface due to formed π − π or CH-π interactions. Crucially, depending on the structure of the polymer, these materials exhibit different selectivity toward specific SWCNT types^11^. Consequently, conjugated polymers may harvest only the desired species from a raw polychiral material. Promising results have been published for poly(9,9-dioctylfluorenyl-2,7-diyl) (PFO) and its derivatives such as poly(9,9-dioctylfluorene-*alt*-1,4-benzo-(2,10,3)-thiadiazole) (F8BT)^12^. Among the evaluated polymers, PFO exhibits superior selectivity for processing small-diameter s-SWCNTs in 0.8‒1.2 nm range. In particular, it offers exceptional performance, enabling monochiral resolution, suspending only (7,5)^13,14^ or (7,3)^15^ SWCNTs under specific conditions.

Despite the advantages related to the CPE approach, the synthesis of conjugated polymers via coupling reactions is difficult to control and optimize. As a result, produced batches commonly have inconsistent molar weights and dispersity indices (*Đ*). This is a serious issue as it determines the selectivity and yield of the CPE of SWCNTs^16^. The most often used methodology to obtain fluorene-based conjugated polymers is the Suzuki polycondensation reaction. It is an A-B or AA-BB type process wherein monomers consisting of diarylbromide moieties and diarylboronate esters are combined. This reaction is commonly catalyzed by Pd complexes such as tetrakis (triphenylphosphine) palladium(0) (Pd(PPh_3_)_4_), which in most cases provides satisfactory yields. Although it is a highly active and efficient homogeneous catalyst, its high price, arduous synthesis route, air sensitivity, and lack of recyclability hinder scale-up opportunities for the production of conjugated polymers and limit their application scope. However, these obstacles can be overcome via the development of new catalytic systems based on modern materials that can retain high activity and selectivity while simultaneously working in the presence of air for a prolonged time.

Certain nanomaterials fulfill the aforementioned conditions. For instance, reports have shown that palladium nanoparticles (PdNPs) have been successfully implemented for Suzuki cross-coupling reaction of aryl halides containing -methyl, -methoxy, and -nitro moieties^17–19^, making them excellent candidates for improving the protocol of conjugated polymer synthesis. However, quasi-homogeneous PdNPs are cumbersome to separate from the reaction mixture after polymerization, which reduces their applicability and increases the process costs. Therefore, to overcome this issue, the provision of a heterogeneous character to the catalytic system is a reasonable solution.

In this study, we prepared nickel nanowires (NiNWs) that acted as a scaffold for the deposition of highly active PdNPs. The nanocomposite catalytic system was used for the Suzuki polymerization of fluorene-based moieties. To the best of our knowledge, this is the first reported use of PdNPs/NiNWs nanocomposite in this application. NiNWs were synthesized via simple wet reduction of metal salts^20^, followed by a sonochemical approach to create PdNPs on their surface^21^. The as-prepared nanocatalyst was employed in conjunction with microwave heating for the synthesis of conjugated polymers based on polyfluorene. The developed method permitted the polymerization process to be significantly accelerated compared to the classical thermal approach^22–25^. The reaction time was considerably shortened from 3 d to only 1 h, which drastically improved the economics of polymer production. The obtained conjugated polymers were then evaluated as selective SWCNT dispersants for sorting polychiral raw material.

## Experimental

A list of reagents and procedures (including synthesis of PdNPs/NiNWs and selective isolation of SWCNTs) and methods for characterization of the materials involved in this study are presented in the SI.

## Results and discussion

### Catalyst characterization

**Figure 1:**
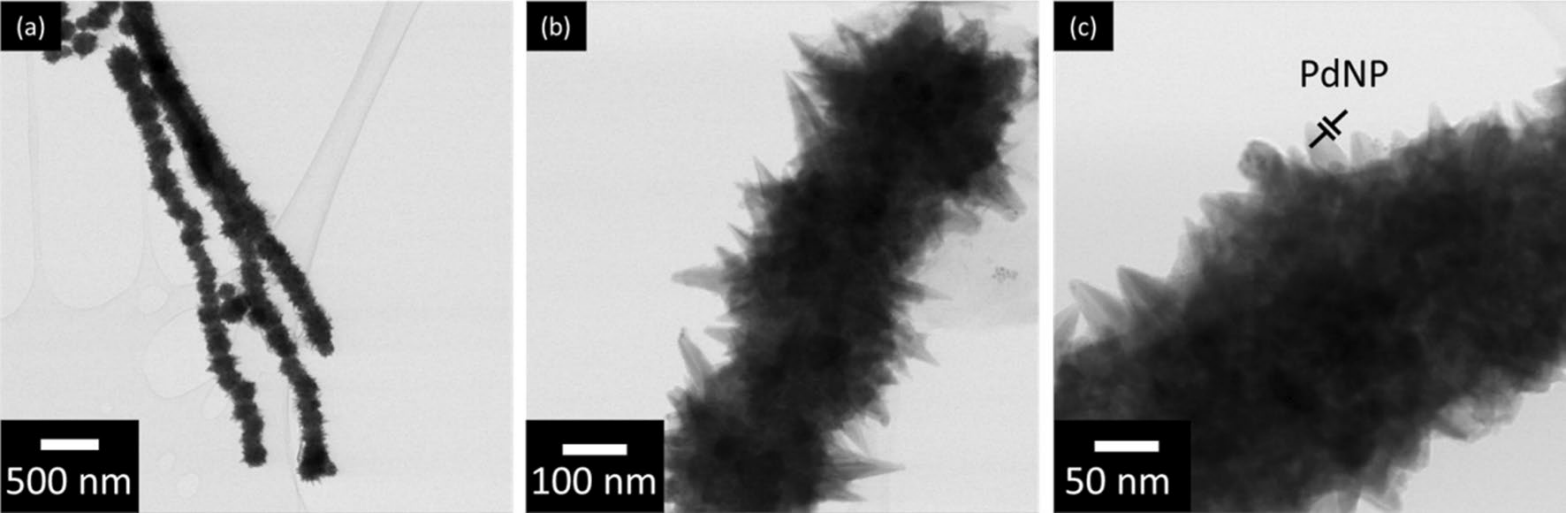
TEM micrographs of PdNPs/NiNWs nanocatalyst at various magnifications.

The support nanomaterials (NiNW) with anisotropic structure were synthesized by simple wet chemical reduction. A magnetic field forced the alignment of paramagnetic Ni^2+^ complexes, which enhanced the step-growth of protonanowire^26^, resulting in relatively long NiNWs (Fig. 1a). The nanomaterials possessed characteristic spikes along an anisotropic nanostructure, which provided a developed surface area (Fig. 1b). Subsequently, a sonochemical approach was engaged for PdNPs deposition. PdNPs were successfully deposited on NiNW support, and their diameters were in the order of the range of single nanometers according to TEM measurements (Fig. 1c). Additionally, ICP-OES was used to determine that Pd concentration was 0.34%, which was employed for calculating TOF values in further catalytic tests.

**Figure 2:**
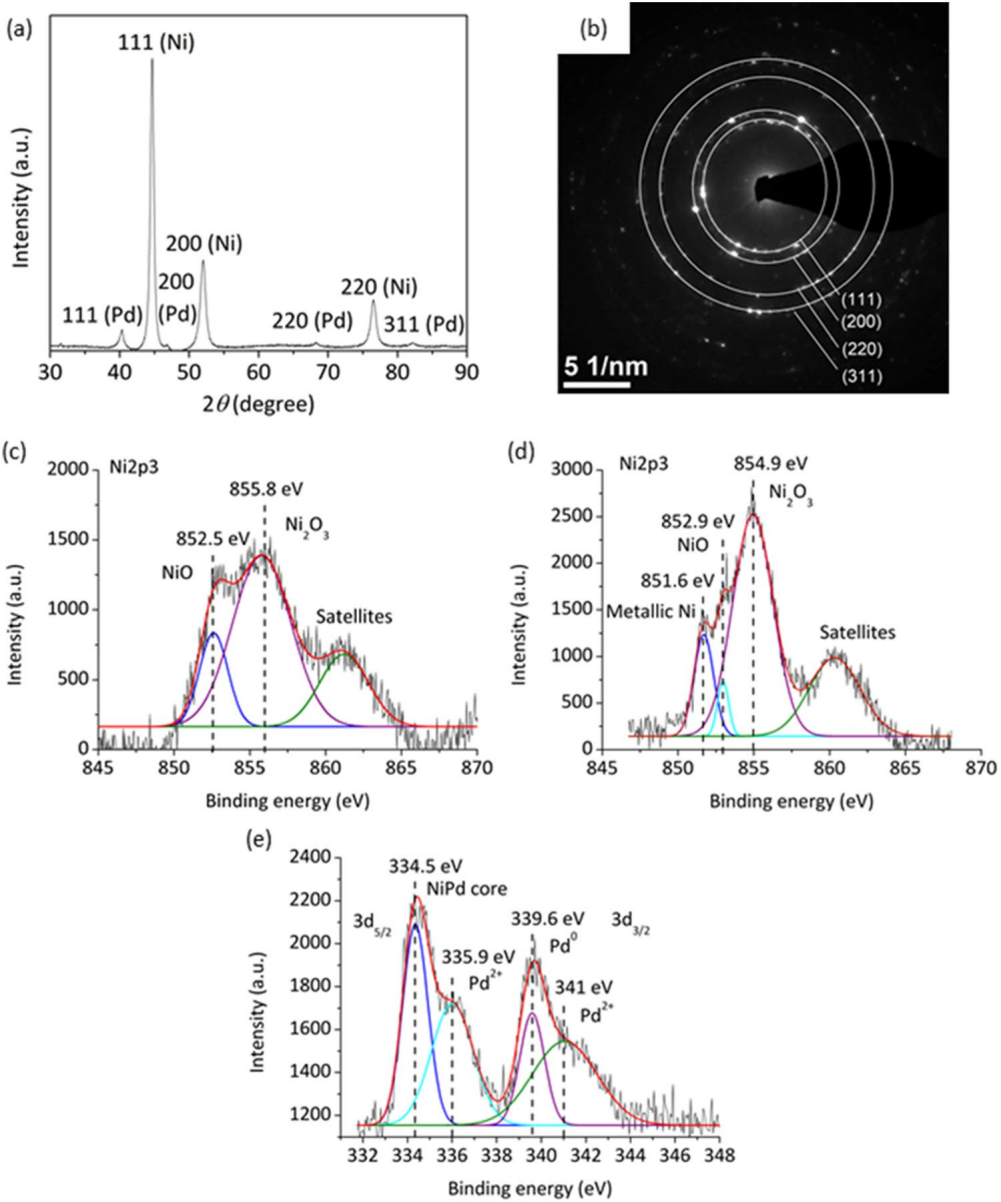
(**a**) XRD patterns of PdNPs/NiNWs nanocatalyst, (**b**) SAED patterns of PdNPs/NiNWs nanocatalyst (**c**) Ni2p_3/2_ of NiNWs, (**d**) Ni2p_3/2_ of Pd/NiNWs (**e**) Pd3d_3/2_ and Pd3d_5/2_ X-ray photoemission spectra recorded for PdNPs/NiNWs sample.

XRD patterns confirmed the presence of Ni and Pd atoms in the material (Fig. 2a). Three features related to Ni at 2θ = 44.6°, 52°, and 76.6° were indexed as (111), (200), and (220) lattice planes, respectively (JCPDS card, File No. 04-0850). They were detected in pure NiNWs before Pd deposition, supporting this assignment (Fig. S4). The peaks at 40.3° (111), 46.8° (200), 68.3° (220), and 82.1° (311) were related to Pd (JCPDS card, File No. 46-1043), which indicated the presence of a face-centered cubic phase. Selected Area Electron Diffraction (SAED) revealed only Ni patterns (111), (200), (220) and (311). The latter was absent in XRD spectra as its location extended beyond the selected examination range. It should appear at ca. 93.0°. The lack of any Pd-related patterns can be explained by a very small concentration of Pd in the sample (less than 0.5%).

XPS analysis provided relevant information about the electronic states of the catalyst surface. At first, bare NiNWs were analyzed. The obtained spectra showed peaks of NiO and Ni_2_O_3_ at 852.5 eV and 855.8 eV, respectively, in Ni2p_3/2_ binding energy band. After Pd deposition, a metallic Ni signal was found at 851.6 eV, and nickel oxide signals increased significantly at 852.9 eV (NiO) and 854.9 eV (Ni_2_O_3_). This can be explained as a result of partial reduction of nickel during sonochemical process and subsequent oxidation in air. Pd showed the binding energies of Pd3d_3/2_ and Pd3d_5/2_. According to the reference values (340.36 eV and 335.1 eV)^27^, PdNPs consisted of Pd(0) (339.6 eV) and Pd^2+^ (335.9 eV, 341 eV). Interestingly, the peak at 334.5 eV could represent Pd atoms included in the NiNW core.

### Conventional heating route

The study of the catalytic properties involved the standardized procedure for Suzuki polymerization of fluorene-based polymers^28^. In brief, both monomers were dissolved in toluene, and then an aqueous solution of sodium carbonate was added. As the reaction mixture consisted of two immiscible liquids, the synthesis occurred in an interfacial region. A few drops of Aliquat 336 were added as a phase-transfer catalyst to enhance the mass transport process. Commercially available Pd(PPh_3_)_4_ catalyst required inert atmospheric conditions, which was achieved by purging the solution with nitrogen before and after the addition of the catalyst. For PdNPs/NiNWs catalyst, this was unnecessary due to its superior stability in the air. In both tests, the solutions were stirred in closed glass reactors at elevated temperatures. The sealed environment allowed for over-pressure generation and condensation of vapors on the glass walls. After 3 d of vigorous stirring to ensure good contact between both liquid phases, the toluene phase containing Pd(PPh_3_)_4_ turned black, suggesting the completion of the reaction. In the case of the PdNPs/NiNWs-driven reaction, the top phase turned a yellow color. Polymerization using a commercial catalyst gave a higher yield (83%) compared to the poor initial performance of PdNPs/NiNWs under conventional heating (16%) on the same time scale. Unfortunately, PdNPs/NiNWs nanocatalysts, under the applied conditions, were only able to form oligomers. The elution volume peaks registered for the samples by GPC were only slightly lower than the solvent peak, suggesting that the product comprised mostly of PFO dimers (M_n_ = 0.909 kg/mol and *Đ* = 1.24). In contrast, the commercial catalyst-driven reaction gave the expected polymer, characterized by M_n_ = 15.61 kg/mol and *Đ* = 3.19. Therefore, while the thermal stimulus was insufficient to activate the PdNPs/NiNWs nanocatalyst, the homogeneous commercial formulation based on Pd(PPh_3_)_4_ was successful.

### Microwave-assisted route

The various desirable effects of microwave radiation resulting from both thermal and non-thermal effects have been consistently reported in organic synthesis^29^, but also directly in the area of Suzuki polycondensation^30^. The irradiation enhances molecular mobility, enabling efficient mixing without the employment of magnetic stirring. This aspect is particularly important owing to the limited performance of PdNPs/NiNWs stemming from the paramagnetic properties of NiNWs. This promotes their accumulation on the surface of the dipole used for stirring the liquid medium in the reactor, possibly reducing the available catalytic surface area. Since, after the first tests, we noticed that the performance achieved by the PdNPs/NiNWs was higher than in analogous reactions supported by conventional heating, hence, we proceeded to optimize the process.

In this study, we focused on Standard (S) and Solid Phase Synthesis (SPS) modes of the employed microwave system using a broad range of experimental conditions. The first one used variable power to obtain the target temperature and maintained it at approx. ± 1 °C. In contrast, during SPS mode, the power was constant, which led to a higher temperature hysteresis (ΔT≈ 5 °C). While the standard protocol provided a comparable intensity of microwave radiation during the process, SPS mode delivered the power in pulses. Experiments conducted under standard and SPS conditions are listed below (Table 1).

**Table 1:** Microwave-assisted PFO synthesis via Suzuki polycondensation at standard and SPS mode.


No	Mode	Power [W]	Base/Conc	Temp. [°C]	Yield [%]	TOF [h^-1^]	M_n_ [kg/mol]	M_w_ [kg/mol]	*Đ*	Degree of polymerization
1	S	0–200	Na_2_CO_3_/1 M	80	0	–	–	–	–	–
2	S	0–200	Na_2_CO_3_/1 M	110	62	1793	1.88	3.06	1.63	5
3	S	0–200	Na_2_CO_3_/1 M	130	57	1648	8.08	11.31	1.40	21
4	S	0–200	K_2_CO_3_/1 M	80	34	983	3.14	3.99	1.27	8
5	S	0–200	K_2_CO_3_/1 M	110	69	1995	5.25	8.45	1.61	14
6	S	0–200	K_2_CO_3_/1 M	130	75	2168	4.56	6.61	1.45	12
7	SPS	80	Na_2_CO_3_/1 M	75–80	0	–	–	–	–	–
8	SPS	80	Na_2_CO_3_/1 M	105–110	82	2371	8.32	16.31	1.96	21
9	SPS	80	Na_2_CO_3_/1 M	125–130	41	1185	4.95	6.68	1.35	13
10	SPS	80	K_2_CO_3_/1 M	75–80	51	1475	3.45	4.59	1.33	9
11	SPS	80	K_2_CO_3_/1 M	105–110	69	1995	6.61	11.90	1.80	17
12	SPS	80	K_2_CO_3_/1 M	125–130	60	1735	6.19	8.66	1.40	16
13	SPS	100	Na_2_CO_3_/1 M	105–110	88	2544	14.32	25.99	1.82	37
14	SPS	120	Na_2_CO_3_/1 M	105–110	78	2255	8.37	12.67	1.51	22

Reaction conditions: 0.12 mmol of diarylbromide, 0.12 mmol of diarylboronate ester, 2 mL of base solution, 2 mL of toluene, 1 drop of Alliquat 336, and 10 mg of PdNPs/NiNWs catalyst. The reaction was carried out for 1 h. The degree of polymerization was estimated by dividing M_n_ by monomer mass (0.388 kg/mol).

The experiments shown in the table showed a significant acceleration of the reaction supported by microwave radiation compared to the reaction catalyzed by Pd (PPh_3_)_4_ in conventional heating. In the latter case, no isolated polymer was formed after 1 h. In addition, for most of the conditions tested, the conversion rate was very high, reaching up to 88% under optimal conditions (typically exceeding 60%). Considering the reduction of these values by losses arriving from product isolation, the performance seemed promising.

The observed reaction acceleration was highly beneficial and may lead to the development of more approachable polymer production. A shorter reaction time (1 h compared to 3 d for conventional heating) can reduce process costs considerably, especially in the long term, due to energy saving. Moreover, we demonstrated that the molecular weight (M_w_) could be controlled through optimized polymerization using the described approach (M_w_ in the range of 3–26 kg/mol), which has been rarely reported for thermally-promoted Suzuki polycondensation reactions using typical homogeneous catalysts. Our investigations started with examining the temperature as one of the main parameters during the polymerization process, as it controlled reaction speed. When sodium salt was used, 80 °C was insufficient for the reaction to occur during both modes (Table 1—entries 1 and 7). No signs of the product were discerned. On the other hand, when the temperature of the process was increased to 110 °C, the conditions allowed for the precipitation of the crude product in methanol, which confirmed the presence of oligomers (Table 1—entry 2).

During the Suzuki polycondensation reaction, the choice of base was essential due to its vital role in the palladium catalytic cycle. Both strong and weak bases are typically utilized in the synthetic process depending on substrate structure and reactivity. Strong bases, such as sodium hydroxide (NaOH) or potassium hydroxide (KOH), are used for the deprotonation of the boronic acid monomer, which is crucial for initiating the coupling reaction^31–33^. Carbonate and phosphate salts are more popular for a wide range of substrates, which are free of steric hindrance. These bases facilitate the formation of quaternized organoboron active species with increased nucleophilicity that react with the Pd catalyst, enabling transmetallation and, thus, the polymerization process^34^. However, weak bases, such as triethylamine (TEA) or pyridine, can neutralize the acid byproduct produced during the reaction. These weak bases prevent side reactions and help maintain the appropriate pH level of the reaction mixture, ensuring the success of the polymerization. Although their performance is less efficient, they allow for the polymerization of some electron-deficient aryl halides at higher temperatures^35^. Consequently, the choice of base can have a significant impact on the M_w_ of PFO during polymerization as it, among other factors, affects the kinetics of the polymerization process. Strong bases promote faster polymerization, leading to polymers with higher M_w_^36–38^. Conversely, weak bases slow down the reaction rate, resulting in polymers of lower molecular weights^34,39^.

In the case of the reaction conducted in standard mode, switching to potassium carbonate was beneficial for the experiments conducted at 80 °C and 110 °C due to a substantial increase in M_w_ values (3.99 and 8.45 kg/mol, respectively, vs. lack of product at 80 °C and 3.06 kg/mol registered for the polymer generated at 110 °C) (Table 1—entries 4 and 5 vs. 1 and 2). Interestingly, a further increase of temperature in this mode to 130 °C was favorable for the reaction using Na_2_CO_3_, as M_w_ was even higher (at the slight expense of the yield) (Table 1—entry 3), the analogous process conducted using K_2_CO_3_ resulted in reduced M_w_ with concurrent yield improvement (Table 1—entry 6).

In SPS mode, both cases (sodium and potassium salts) showed a decrease in PFO molecular weights at high process temperatures, especially for sodium carbonate. Additionally, examination of an increase in power while maintaining the same temperature range of 105–110 °C, found that the best results were obtained for 100 W settings (M_w_ of 25.99 kg/mol, Table 1—entry 13). A lower power of 80 W was insufficient to elongate the polymers to the appreciable extent (M_w_ of 11.9 kg/mol, Table 1—entry 11). In comparison, a higher power of 120 W did not provide sustainable conditions for the polymer chain growth (M_w_ of 12.67 kg/mol, Table 1—entry 14). However, in this case, the dispersity decreased from 1.8 to 1.51, which resulted in a slightly higher degree of polymerization (22 vs. 17).

**Figure 3:**
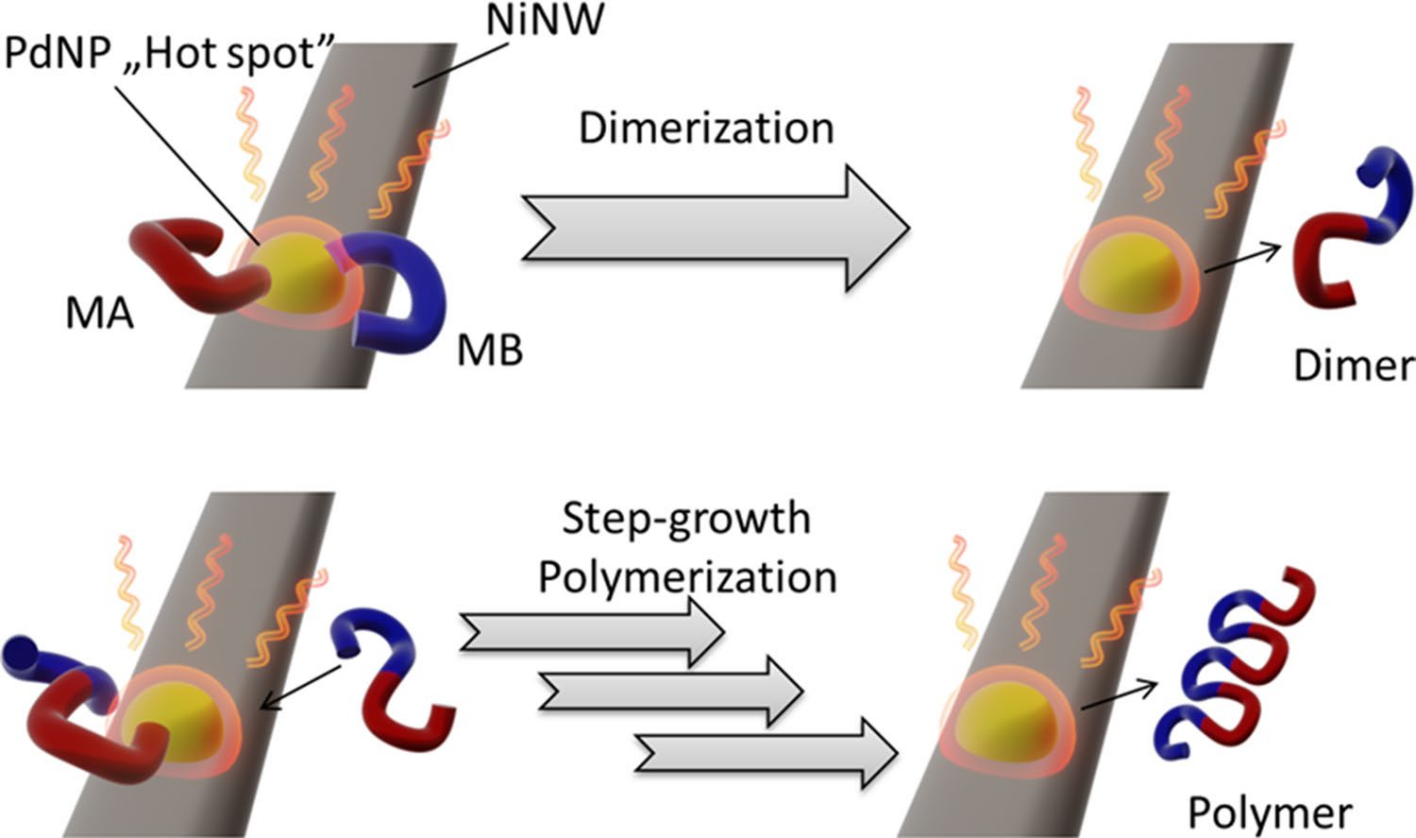
Proposed mechanism of “hot spot” mediated step-growth Suzuki polymerization. MA and MB stand for Monomer A (diarylbromide) and Monomer B (diarylboronate ester), respectively.

Regarding the impact of the microwave settings on the characteristics of the synthesized polymers, SPS mode provided pulses of constant power in short bursts. This possibly led to the generation of hot spots on the metallic nanostructures upon absorption of radiation^40–42^, which improved the diffusion of reactants and provided more energy to overcome the thermodynamic barriers of the process. The obtained results confirmed this reasoning due to longer chain polymers being generated using this mode (Table 1—entries 8 and 11). On the contrary, in standard mode, the microwave power was tuned to reach the desired temperature. Hence, the power ranged between 5 and 15 W to maintain the temperature, and over most of the reaction time the reactor provided only a small portion of radiation, which in turn minimized the hot spot effect. Consequently, the diffusion of reactants to reach the catalytic sites (in the absence of mixing) was hindered. This was detrimental to the reaction rate, which lowered the final molar mass of the polymer, especially in the case of entry 2 in Table 1. As mentioned earlier, microwaves accelerate the process in many ways. The lack of temperature gradient in the reaction vessel and faster heating provided more suitable conditions for the reaction. We believed that Pd hot spots acted as welding spots for joining monomers together, which promoted dimerization and further step-growth polymerization (Fig. 3). Therefore, microwaves accelerated the formation of the studied polymers. Finally, regardless of the microwave heating mode, the synthesized PFO polymers were rather homogeneous as *Đ*, describing the heterogeneity of polymer mass, ranging between 1.27 and 1.96 (the lower values corresponded to better control over polymer chain growth during the synthesis). A step-growth polymerization is often characterized by *Đ* = 2^43^, so the newly designed approach matched the expectations while substantially shortening the reaction times.

### SWCNTs polymer wrapping

One of the intriguing properties of PFO is its high affinity (7,5)-chirality SWCNTs in organic solvents and, thus, its ability to selectively isolate this SWCNT type^12,14,16,44,45^. When PFO is dissolved in an organic solvent, preferably in nonpolar toluene, it forms a complex with (7,5) SWCNT through π-π stacking interactions^46^. The unique arrangement of atoms in (7,5) SWCNT structure facilitates strong and specific binding with PFO^47^. Consequently, this polymer enables selective isolation of (7,5) SWCNTs from polychiral mixtures. Despite the remarkable selectivity of PFO towards (7,5) SWCNT, challenges remain. The interaction between PFO and SWCNT is influenced by various parameters, such as polymer characteristics and selection of solvent^14^ and extraction conditions^48^. Understanding the role of these parameters is crucial for optimizing the selective isolation process.

**Figure 4:**
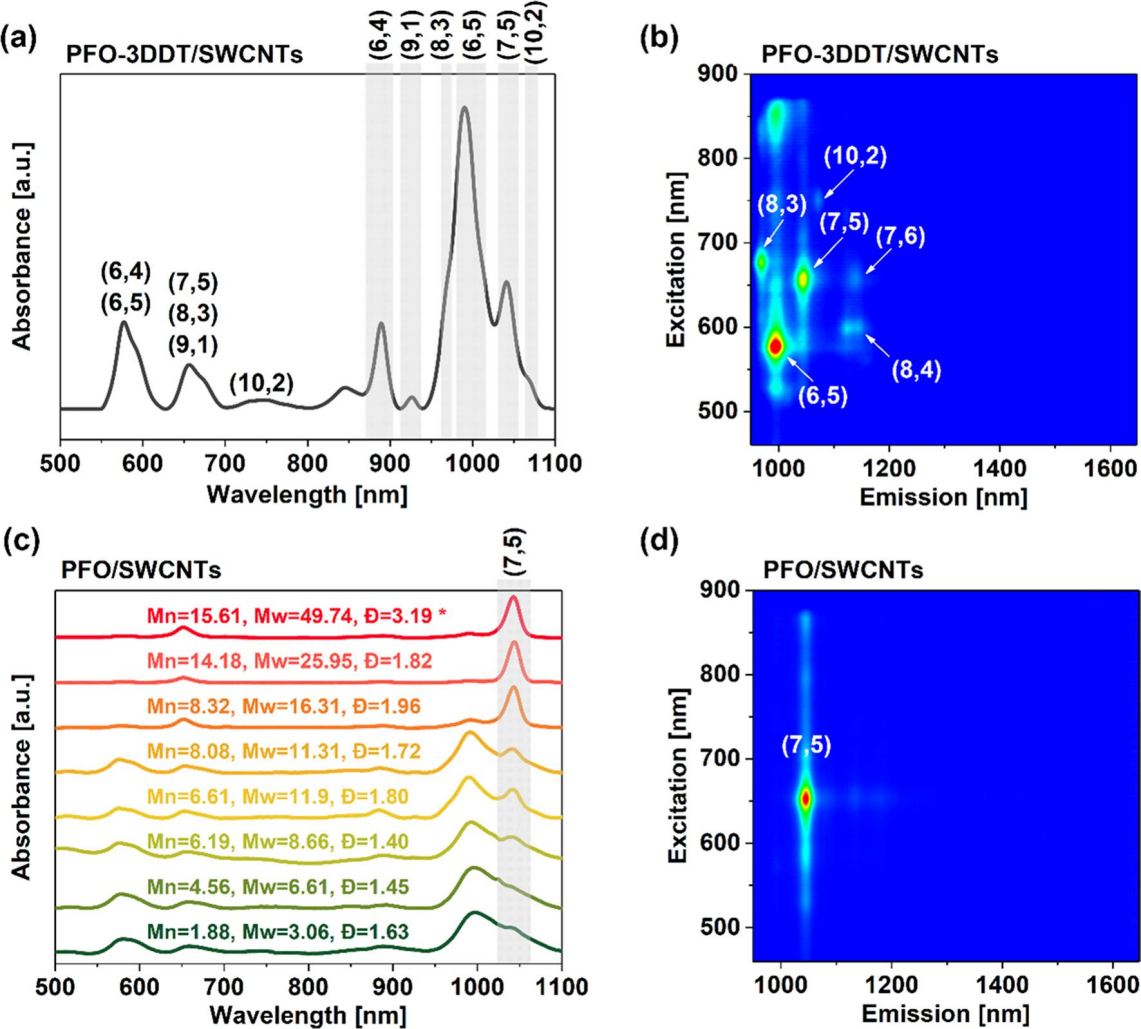
(**a**) UV–VIS spectrum of SWCNTs suspended by PFO-3DDT. (**b**) Excitation-emission PL map of SWCNTs suspended by PFO-3DDT ((6,4) and (9,1) SWCNTs are not visible as they emit outside of the measurement range). (**c**) The influence of molecular characteristics of the synthesized PFO batches on the optical absorbance spectra of SWCNTs suspended with PFO. *The polymer with the highest M_w_ (49.74 kg/mol) refers to Pd(PPh_3_)_4_–driven reaction, which is shown here as a reference. All the other polymers were obtained using PdNPs/NiNWs nanocatalyst. (**d**) Excitation-emission PL map of extracted (7,5) SWCNTs using PFO synthesized using the specified conditions (Table 1, entry 13).

To address the above-mentioned challenges, first, we analyzed the composition of the raw SWCNT mixture by suspending it non-selectively using poly (9,9-dioctylfluorenyl-2,7-diyl-alt-3-dodecylthiophene-2,5-diyl) (PFO-3DDT) conjugated polymer in toluene. The obtained dispersion was studied using optical absorption spectroscopy and photoluminescence (PL) excitation-emission mapping (Fig. 4a,b). The exact positions of the characteristic bands from S_11_ transitions of specific SWCNTs were previously reported in the literature^15^. The dominant chirality in the starting material was (6,5). The amount of (7,5) SWCNTs was also sufficiently high to justify its isolation. This is important because, to date, there is no commercially available raw soot enriched in this particular chirality. In this research, after synthesizing a broad range of PFO polymers, we focused on investigating the influence of M_W_ of PFO on its preference for (7,5) SWCNT wrapping. To this end, the polymers were tested using the model CPE process described in detail in SI. By varying M_W_ of PFO, we found that it played a significant role in the selectivity of the isolation process. The shape of the peaks (their full width at half maxima) and the peak-to-valley ratios^49–52^ determined the quality of SWCNT wrapping. In the case of low-molecular-weight polymers, the spectra were not sufficiently sharp, excluding the possibility of confirming the identity of peaks arising from specific chiralities. Hence, the polymer could not properly individualize SWCNTs, therefore, SWCNT bundles were present in the suspension instead. Furthermore, polymers with an average M_w_ above 8 kg/mol exhibited stronger interactions with (7,5) SWCNTs (Fig. 4c), resulting in enhanced selectivity for this SWCNT type. This was manifested by the appearance of S_11_ transition at 1040 nm. Concomitantly, the sharper shape of the peaks, indicative of SWCNT debundling, confirmed that their individualization was crucial to unlocking the possibility of material purification. A further increase of the polymer M_w_, steadily increased the ratio of (7,5) SWCNT peak to that of different species. At M_w_ of approx. 16 kg/mol, a critical polymer chain length was reached to promote satisfactory separation results since only (7,5) SWCNTs were suspended. The process of CPE involves dynamic adsorption and desorption of polymer chains on/from the SWCNT surface. To achieve successful isolation, a polymer must interact favorably only with a single SWCNT type sufficiently to remain on its surface during sonication and subsequent centrifugation. In our case, we determined that once the polymer reached this threshold M_w_, its adsorption rate on (7,5) SWCNTs significantly exceeded the tendency to desorb (or the desorption energy was higher than that provided by sonication). Either way, the PFO chains gradually accumulated on the (7,5) SWCNT surface reducing the amount of available for other chiralities. As a result, other SWCNTs wrapped to a smaller extent were very unstable in solution and susceptible to reaggregation during centrifugation. Hence, the amount of various chiralities in dispersion decreased rapidly. As shown in the excitation-emission PL map of suspension obtained by sonicating in-house made PFO and raw SWCNTs in toluene, the SWCNT dispersion had a monochiral character (Fig. 4d).

## Conclusions and future perspectives

We successfully developed a PdNPs/NiNWs nanocomposite catalytic system, which was highly active in polymerization by Suzuki coupling. We obtained PFOs with quantitative efficiency with a wide range of M_w_, which helped gain insight into nanoscale phenomena such as the CPE process. A microwave-assisted synthesis reduced the reaction time from 3 d to 1 h, thereby markedly improving the economics of the process. The microwave heating also provided optimum distribution of energy to reaction volume, because of which the characteristics of the products were more favorable. Dispersity indices of less than 2 were achieved for all obtained polymers. Optimization of power, base, temperature, and time resulted in the production of high-quality polymers, which were employed in SWCNT purification. Our findings revealed a direct correlation between the length of polymer chains and the selectivity towards desired chirality, with longer chains demonstrating higher selectivity. Intriguingly, we identified a specific M_w_ threshold, notably at 16.30 kg/mol, corresponding to 20 dimers units for PFO, which gave rise to significant improvement of SWCNT selectivity. Overall, the obtained results pave the way for the design of polymers with considerable application opportunities in materials science, with already documented high utility for purifying SWCNTs.

### Supplementary Information

Supplementary Information.

## Data Availability

The raw/processed data required to reproduce these findings cannot be shared at this time as the data also forms part of an ongoing study.
