# Supplementary material for: PdNPs/NiNWs as a welding tool for the synthesis of polyfluorene derivatives by Suzuki polycondensation under microwave radiation
Source: Sci Rep. 2024 Jan 28;14:2336. doi: 10.1038/s41598-024-52795-w (PMC10822865; doi:10.1038/s41598-024-52795-w)
Supplement: Supplementary file 1 — Supplementary Information. [file 41598_2024_52795_MOESM1_ESM.docx]

**Supplementary Information**

**PdNPs/NiNWs as a welding tool for the synthesis of polyfluorene derivatives by Suzuki polycondensation under microwave radiation**

Tomasz Wasiak^1^, Dominik Just^1^, Andrzej Dzienia^1^, Dariusz Łukowiec^2^, Stanisław Wacławek^3^,
Anna Mielańczyk^1^, Sonika Kodan^4^, Ananya Bansal^4^, Ramesh Chandra^4^, Dawid Janas^1,*^

*^1^ Department of Chemistry, Silesian University of Technology, B. Krzywoustego 4, 44-100, Gliwice, Poland*

*^2^ Materials Research Laboratory, Faculty of Mechanical Engineering, Silesian University of Technology, Konarskiego 18a, 44-100, Gliwice, Poland*

*^3^ Institute for Nanomaterials, Advanced Technologies and Innovation, Technical University of Liberec, Studentská 1402/2, 461 17 Liberec 1, Czech Republic*

*^4^ Nanoscience Laboratory, Institute Instrumentation Centre, Indian Institute of Technology Roorkee, Roorkee-247667*

1. **Materials and equipment**

The following chemicals were used as-received without any further purification: NiCl_2_∙6H_2_O (Acros Organics, Belgium, 97%), NaOH (Chempur, Poland, pure p.a.), polyvinylpyrrolidone (PVP; Alfa Aesar, molecular weight 1.3 MDa), ethylene glycol (EG; Chempur, Poland, pure p.a.), KCl (Alfa Aesar, 99%), PdCl_2_ (ReagentPlus; Sigma-Aldrich, 99%), Alliquat 336 (Alfa Aesar), methanol (PureLand, Poland, 99.85% pure p.a.), acetone (PureLand, Poland, 99.6% pure p.a.), toluene (Alfa Aesar, spectrophotometric grade, +99.7%), Na_2_CO_3_ (Acros Organics, anhydrous, extra pure, 99.5%), K_2_CO_3_ (Chempur, pure p. a.), chloroform (P.P.H. STANLAB, pure p. a.), MgSO_4_ (Chempur, anhydrous pure p. a.), N_2_H_4_∙H_2_O (Alfa Aesar, 98%, pure p.a.), 9,9-dioctylfluorene-2,7-bis(boronic acid pinacol ester) (Angene, 98%), 2,7-dibromo-9,9-dihexyl-9H-fluorene (AmBeed, 97%), 9,9-dioctyl-2,7-dibromofluorene (Sigma Aldrich, 95%), and tetrakis (triphenylphosphine)palladium - Pd(PPh_3_)_4_, (Apollo Scientific, 99%). To evaluate the synthesized polymers, Conjugated Polymer Extraction (CPE) processes were conducted on CoMoCAT SWCNTs (SG65i, Signis; purity: 95%-carbon basis).

1. **Synthesis of NiNWs**

The synthetic procedure was based on previously published work [1]. Briefly, 70 mL of 0.1 M NaOH_(EG)_ was poured into a 600 mL beaker and mixed with 5 mL of 64% hydrazine hydrate_(aq)_. The mixture was heated to 90 °C. In a separate beaker (100 mL), a neodymium magnet covered with a paper towel was placed as the magnetic field source. The beaker containing the magnet was placed into the beaker containing the reaction solution to provide a magnetic field for the anisotropic growth of NiNWs. Then, 20 mL of NiCl_2(EG)_ was slowly added dropwise into the solution. A precipitate appeared and turned from a blue to black color in a few seconds. The formation of a black sponge-like solid continued for 10 min. The obtained NiNWs were separated by a magnet, washed three times with distilled water and three times with acetone, dried in air, and stored in a desiccator.

1. **Decorating NiNWs with PdNPs**

KCl and PdCl_2_ in 2:1 mol ratio were added to 100 mL of deionized water in a three-necked round-bottom flask to generate a 5 mM K_2_PdCl_4_ salt solution. This solution was mixed for 1 h before the addition of 100 mg of PVP, followed by vigorous stirring until full dissolution was achieved. Then, 120 mg of NiNWs was added to the solution, and the flask was purged with N_2_ gas for 10 min before being placed in an ultrasonic water bath (Bandelin Sonorex RK 102H, 320 W, 35 kHz) heated to 60 °C for 1 h under an inert atmosphere. After the deposition of PdNPs on NiNWs was complete, the product was separated by a magnet and rinsed with copious amounts of distilled water, washed three times with acetone, and then air dried. The obtained PdNPs/NiNWs catalyst was stored in a desiccator.

1. **Suzuki polymerization promoted by conventional heating**

The reactions were carried out in a glass reactor sealed with a Teflon cap. 0.19 mmol of diarylbromide (MA), 0.19 mmol of diarylboronate esters (MB), 5 mmol Na_2_CO_3_, 5 mL of toluene, 5 mL of deionized water, 3 drops of Alliquat 336, and a stirring bar were placed inside the reactor and purged with N_2_. Then, 2% mol Pd(PPh_3_)_4_ was added, and the mixture was purged with N_2_ once again. For PdNPs/NiNWs–driven reactions, 10 mg of nanocatalyst was added instead of Pd(PPh_3_)_4_. The reactor was sealed and placed in an oil bath heated to 85 °C. The reactions were conducted for 3 days. After the reaction was complete, the mixture was extracted with CHCl_3_, washed with water and brine, and dried over MgSO_4_. The obtained mixture was concentrated in vacuo, dissolved in a small portion of CHCl_3_, and poured dropwise into vigorously stirred cold methanol. The formed precipitate was collected by filtration, washed with methanol and acetone, and air dried.

1. **Suzuki polymerization in a microwave reactor**

The reaction was carried out in a glass tube closed with a Teflon seal. 0.12 mmol of diarylbromide, 0.12 mmol of diarylboronate esters, 2 mmol Na_2_CO_3_ or K_2_CO_3_, 2 mL of toluene, 2 mL of deionized water, and 1 drop of Alliquat 336 were placed in a glass tube, and the mixture was purged with N_2_. Then, 2% mol Pd(PPh_3_)_4_ was added, and the mixture was purged with N_2_  once again. For PdNPs/NiNWs-driven reactions, 10 mg of nanocatalyst was added instead of Pd(PPh_3_)_4_. The tube was sealed, vortexed, and placed in the microwave reactor unit (CEM Discover SP). The reaction was carried out under the conditions described in Table 1 for 1 h. The product was isolated, purified, and characterized using the same procedures described in the previous section for the conventional method.

1. **Procedure for conjugated polymer wrapping**

The dispersion process was conducted as follows, 1.5 mg of SWCNT ((6,5)-enriched CoMoCAT SWCNTs) and 9 mg of polymer (ratio of polymer to SWCNTs = 6:1) were weighed into two 13 mL glass vials. The polymer was then dissolved in 5 mL of toluene at room temperature in one vial. The as-prepared polymer solution was transferred to the second vial with pre-weighed SWCNTs. The mixture was sonicated under mild conditions, in an ice-cooled bath sonicator for 15 min (POLSONIC, SONIC-2, 250 W). The next step was to carry out main the sonication process using a tip sonotrode (Hielscher UP200St ultrasonic generator) to debundle the SWCNTs and wrap them with polymer chains. During this step, more energy was supplied to the system (the tip of the sonotrode operated at 30 W for 8 min at approx. 0 °C). After sonication, the generated slurry was transferred to 15 mL conical tubes and centrifuged at 10,000 rpm (15,314 x g) for 5 min to remove unwrapped SWCNTs and polymer aggregates. Finally, the collected supernatant was transferred to a fresh vial and analyzed.

1. **Materials characterization**
   1. PdNPs/NiNWs

Morphology studies were carried out by Scanning-Transmission Electron Microscopy (STEM, S/TEM TITAN 80-300) and Transmission Electron Microscopy (TEM, S/TEM TITAN 80-300). Energy-dispersive X-ray spectra (EDS) and Selected Area Electron Diffraction (SAED) patterns were collected using the specified TEM. The elemental compositions of PdNPs/NiNWs catalysts were determined by ICP-OES (PerkinElmer, Optima 2100 DV), calibrated with commercially available standards provided by Sigma-Aldrich company. X-ray diffraction patterns were collected by Bruker AXS model D8 using λ_Cukα_ = 0.154 nm and applied power of 40 kV and 40 mA. XPS measurements were performed using PHI 5000 Versa Probe III with monochromatic Al_Kα_ source and 1486.7 eV energy at 10^-7^ mbar pressure and 55 eV pass energy.


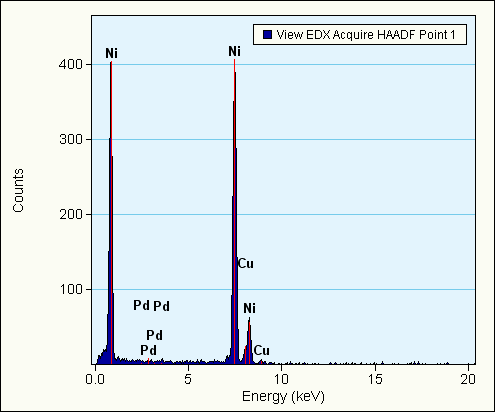


**Fig. S1.** EDS spectrum of PdNPs/NiNWs nanocatalyst confirming the presence of Pd and Ni.

- 1. Conjugated polymers

NMR spectra were recorded at 298 K on an Agilent-MR NMR (Palo Alto, CA, USA; 400 MHz). In all cases, CDCl_3_ was used as the solvent and trimethylsilane as an internal reference. Size exclusion chromatography (SEC) was employed to determine molecular weights and polydispersity indices of synthesized polymers (1100 Agilent 12060 Infinity. Linear polystyrene standards (580‒300,000 g/mol) were used for calibration. PLGel 5µm MIXED-C 300 × 7.5 mm column connected to precolumn guard (5 µm, 50 × 7.5 mm) was used. Polymers were dissolved in HPLC-grade dichloromethane, and the measurements were conducted at 30 °C and 0.8 mL/min flow rate.


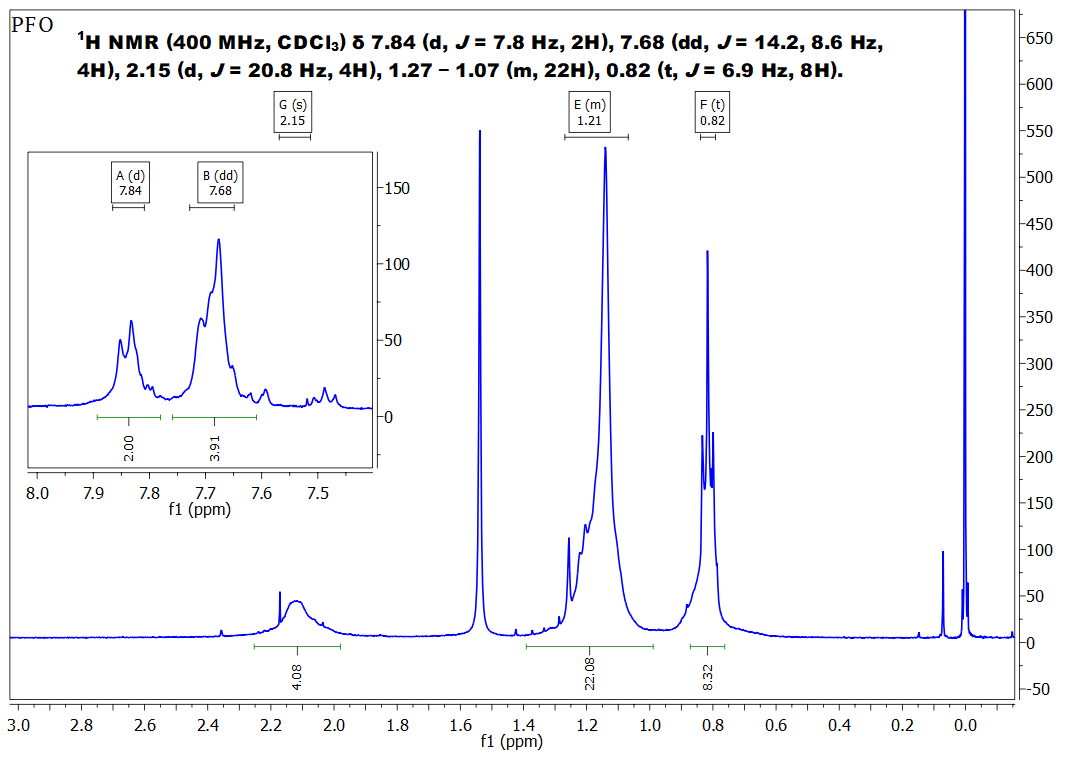


**Fig. S2.** ^1^H NMR spectrum of PFO (Table 1 – entry 14).

**Fig. S3.** GPC chromatograms of obtained PFO batches. Reference material stands for PFO synthesized under conventional heating conditions using Pd(PPh_3_)_4_ as the catalyst.


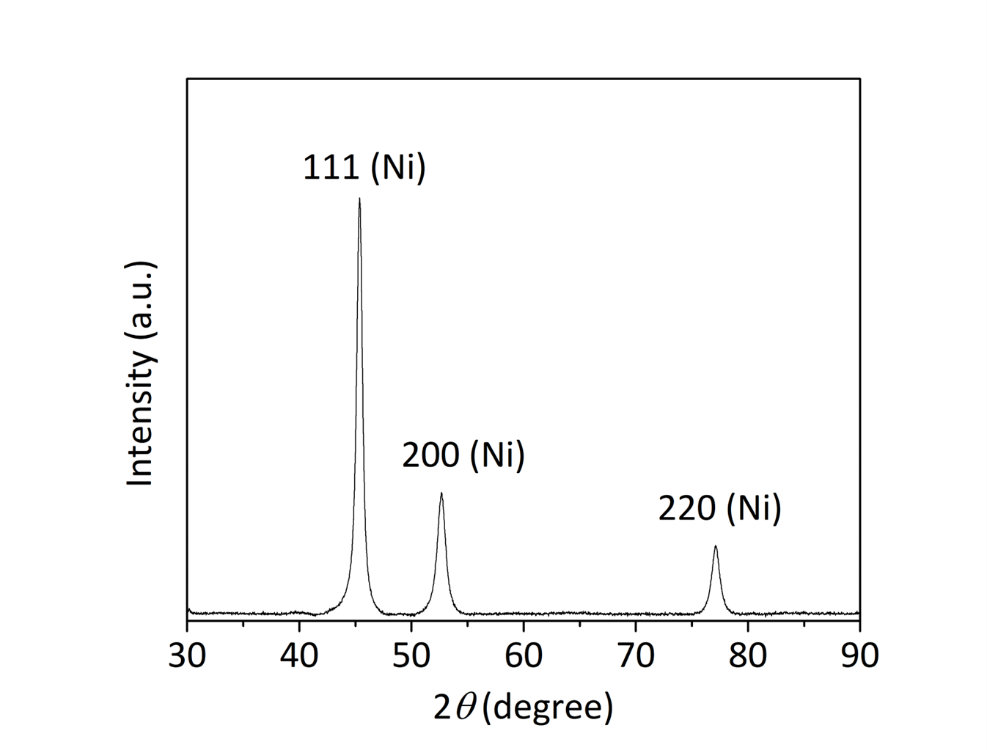


**Fig. S4.** XRD patterns of NiNWs before Pd deposition.

- 1. **SWCNTs dispersions**

UV-Vis spectra were recorded on a Hitachi U-2910 UV-VIS spectrophotometer in a wavelength range of 280‒1100 nm. Asymmetric least squares smoothing (ALSS) was applied for background removal using OriginPro 2022 software, and data normalization in a range of 0 to 1 was used to facilitate qualitative comparison of the recorded spectra [2,3]. Excitation-emission photoluminescence (PL) maps were registered in 480‒900 nm ranges for excitation and 900‒1600 nm for emission by ClaIR microplate reader (Photonetc, Canada). Data was visualized using OriginPro 2022 software.

1. **References**

[1] T. Wasiak, L. Przypis, K. Walczak, D. Janas, Nickel Nanowires: Synthesis, Characterization and Application as Effective Catalysts for the Reduction of Nitroarenes, Catalysts. 8 (2018) 566. https://doi.org/10.3390/catal8110566.

[2] Origin Help - ALS Baseline, (n.d.). https://www.originlab.com/doc/Origin-Help/PeakAnalyzer-ALSBaseline.

[3] Y. Maeda, Y. Konno, A. Nishino, M. Yamada, S. Okudaira, Y. Miyauchi, K. Matsuda, J. Matsui, M. Mitsuishi, M. Suzuki, Sonochemical reaction to control the near-infrared photoluminescence properties of single-walled carbon nanotubes, Nanoscale. 12 (2020) 6263–6270. https://doi.org/10.1039/D0NR00271B.
